# Supplementary material for: COZOID: contact zone identifier for visual analysis of protein-protein interactions
Source: BMC Bioinformatics. 2018 Apr 6;19:125. doi: 10.1186/s12859-018-2113-6 (PMC5889581; doi:10.1186/s12859-018-2113-6)
Supplement: Supplementary file 2 — Software build. Executable binary file of the software tool. (ZIP 118,784 kb) [file 12859_2018_2113_MOESM2_ESM.zip › cozoid/platform/docs/testng-6.5.1-javadoc.zip!/org/testng/annotations/IConfigurationAnnotation.html]

IConfigurationAnnotation (TestNG)


---


|  |  |  |
| --- | --- | --- |
| **Overview** \| **Package** \| **Class** \| **Tree** \| **Deprecated** \| **Index** \| **Help** | |  |
| **PREV CLASS**   **NEXT CLASS** | **FRAMES**    **NO FRAMES**     **All Classes** |  |
| SUMMARY: NESTED \| FIELD \| CONSTR \| METHOD | DETAIL: FIELD \| CONSTR \| METHOD |  |


---


## org.testng.annotations Interface IConfigurationAnnotation

**All Superinterfaces:**: IAnnotation, IParameterizable, ITestOrConfiguration

---

``` public interface IConfigurationAnnotation extends ITestOrConfiguration ```

Encapsulate the @Configuration / @testng.configuration annotation
Created on Dec 20, 2005

**Author:**
:   Cedric Beust

---

| **Method Summary** | |
| --- | --- |
| `java.lang.String[]` | `getAfterGroups()`             The list of groups that this configuration method will run after. |
| `boolean` | `getAfterSuite()`             If true, the annotated method will be run after all tests in this suite have run. |
| `boolean` | `getAfterTest()`             If true, the annotated method will be run after all every test. |
| `boolean` | `getAfterTestClass()`             If true, the annotated method will be run after all the tests in the test class have been run. |
| `boolean` | `getAfterTestMethod()`             If true, the annotated method will be run after any test method is invoked. |
| `boolean` | `getAlwaysRun()`             Used only for after type of configuration methods. |
| `java.lang.String[]` | `getBeforeGroups()`             The list of groups that this configuration method will run before. |
| `boolean` | `getBeforeSuite()`             If true, the annotated method will be run before this suite starts. |
| `boolean` | `getBeforeTest()`             If true, the annotated method will be run before every test |
| `boolean` | `getBeforeTestClass()`             If true, the annotated method will be run after the test class is instantiated and before the test method is invoked. |
| `boolean` | `getBeforeTestMethod()`             If true, the annotated method will be run before any test method is invoked. |
| `boolean` | `getInheritGroups()`             If true, this @Configuration method will belong to groups specified in the \@Test annotation on the class (if any). |
| `boolean` | `isFakeConfiguration()`             Internal use only. |

**Methods inherited from interface org.testng.annotations.ITestOrConfiguration**

`getDependsOnGroups, getDependsOnMethods, getDescription, getGroups, getTimeOut, setDependsOnGroups, setDependsOnMethods, setDescription, setGroups, setTimeOut`

**Methods inherited from interface org.testng.annotations.IParameterizable**

`getEnabled, getParameters, setEnabled`

**Method Detail**

### getBeforeTestClass

```
boolean getBeforeTestClass()
```

:   If true, the annotated method will be run after the test class is instantiated
    and before the test method is invoked.

---


### getAfterTestClass

```
boolean getAfterTestClass()
```

:   If true, the annotated method will be run after all the tests in the test
    class have been run.

---


### getBeforeTestMethod

```
boolean getBeforeTestMethod()
```

:   If true, the annotated method will be run before any test method is invoked.

---


### getAfterTestMethod

```
boolean getAfterTestMethod()
```

:   If true, the annotated method will be run after any test method is invoked.

---


### getBeforeSuite

```
boolean getBeforeSuite()
```

:   If true, the annotated method will be run before this suite starts.

---


### getAfterSuite

```
boolean getAfterSuite()
```

:   If true, the annotated method will be run after all tests in this suite
    have run.

---


### getBeforeTest

```
boolean getBeforeTest()
```

:   If true, the annotated method will be run before every test

---


### getAfterTest

```
boolean getAfterTest()
```

:   If true, the annotated method will be run after all every test.

---


### getAlwaysRun

```
boolean getAlwaysRun()
```

:   Used only for after type of configuration methods. If set to true than
    the configuration method will be run whatever the status of before
    configuration methods was.

---


### getInheritGroups

```
boolean getInheritGroups()
```

:   If true, this @Configuration method will belong to groups specified in the
    \@Test annotation on the class (if any).

---


### getBeforeGroups

```
java.lang.String[] getBeforeGroups()
```

:   The list of groups that this configuration method will run before.

---


### getAfterGroups

```
java.lang.String[] getAfterGroups()
```

:   The list of groups that this configuration method will run after.

---


### isFakeConfiguration

```
boolean isFakeConfiguration()
```

:   Internal use only.

    :   **Returns:**: true if this configuration annotation is not a "true" configuration annotation but a @BeforeSuite or similar that is represented as a configuration annotation.


---


|  |  |  |
| --- | --- | --- |
| **Overview** \| **Package** \| **Class** \| **Tree** \| **Deprecated** \| **Index** \| **Help** | |  |
| **PREV CLASS**   **NEXT CLASS** | **FRAMES**    **NO FRAMES**     **All Classes** |  |
| SUMMARY: NESTED \| FIELD \| CONSTR \| METHOD | DETAIL: FIELD \| CONSTR \| METHOD |  |


---
